# A new family-based association test via a least-squares method

**DOI:** 10.1186/1471-2156-6-S1-S110

**Published:** 2005-12-30

**Authors:** Song Yang, Jungnam Joo, Ziding Feng, Jing-Ping Lin

**Affiliations:** 1 Office of Biostatistics Research, National Heart, Lung, and Blood Institute, Bethesda, Maryland 20892, USA; 2 Cancer Prevention and Research Program, Fred Hutchinson Cancer Research Center, Seattle, WA 98109, USA

## Abstract

To test the association between a dichotomous phenotype and genetic marker based on family data, we propose a least-squares method using the vector of phenotypes and their cross products within each family. This new approach allows covariate adjustment and is numerically much simpler to implement compared to likelihood- based methods. The new approach is asymptotically equivalent to the generalized estimating equation approach with a diagonal working covariance matrix, thus avoiding some difficulties with the working covariance matrix reported previously in the literature. When applied to the data from Collaborative Study on the Genetics of Alcoholism, this new method shows a significant association between the marker rs1037475 and alcoholism.

## Background

Case-control studies provide an important tool to test for the association between disease outcomes and genetic markers [1,2]. Family-based association studies take advantage of existing data, such as data from a previous linkage analysis [3,4].

Incorporation of covariates into the analysis should increase the power to detect associations. However, within-family correlations must be considered. For this purpose the generalized estimating equation (GEE) approach [5] is often used. In the case of dichotomous phenotypes, the GEE approach usually specifies that the mean response is related to a set of covariates via a link function. As for the correlation, usually a common correlation is assumed for each pair of relatives in the working correlation matrix, although more accurate correlation structures are possible and may be more efficient. However, a common problem of GEE is that the working correlation matrix may be singular. Recently Slager et al. [6] showed in various simulation studies that the failure rate for the GEE could be quite high in some cases, and should not be ignored. To remedy this problem they proposed a score test approach for tests of association.

In this article we propose a new association test and apply it to the data from Collaborative Study on the Genetics of Alcoholism (COGA). This new test is derived from a least-squares approach in which the dichotomous responses and their cross products are used, rather than the usual procedure in which the estimating equations only use the observed responses themselves. This approach is asymptotically equivalent to a GEE approach with a diagonal working correlation matrix, and therefore the estimating equation is always well defined.

## Methods

Let *y*_*ij *_be the dichotomous phenotype from the *j*^th ^individual of the *i*^th ^family, where there are *k*_*i *_members from the *i*^th ^family and *n *families in the sample. Let *x*_*ij *_be the covariate vector, decomposed as *x*_*ij *_= (*x*_*ijm*_, *x*_*ije*_), where *x*_*ijm*_, *x*_*ije *_represent the marker allele effect and measured covariates respectively. Suppose that, for the *i*^th ^family, the phenotypes are conditionally independent given a common random effect *u*_*i*_, where the *u*_*i *_values are independent and identically distributed with gamma distribution with mean 1 and variance *θ *> 0, and that, given *u*_*i *_and *x*_*ij*_,

*P*(*y*_*ij *_= 1| *x*_*ij*_, *u*_*i*_) = exp(-*u*_*i*_exp(*x*_*ij*_*β*)), *j *= 1, ..., *k*_*i*_, *i *= 1, ..., *n*,   (1)

where *x*_*ij*_*β *= *x*_*ijm*_*β*_*m *_+ *x*_*ije*_*β*_*e*_. From this, we obtain that the mean of *y*_*ij *_is {1 + *θ *exp(*x*_*ij*_*β*)}^-1/*θ*^. Numerically, it is more stable to work with the reparametrization *η *= *log*(*θ*). In this reparametrization, the mean of *y*_*ij *_is

*P*(*y*_*ij *_= 1) = *A*_*ij *_(*η*, *β*), *j *= 1, ..., *k*_*i*_, *i *= 1, ..., *n*,   (2)

where *A*_*ij *_= {1 + exp(*η *+ *x*_*ij*_*β*)}^-exp(-*η*)^. The joint distribution of *Y*_*i *_= (*y*_*i*1_, ..., ) can also be obtained by integrating out the random effect *u*_*i *_and thus the likelihood function has a closed form. This is an appealing and important feature of the above modelling approach when using the log-log link function and log-gamma random effect. In comparison, for dealing with correlated dichotomous responses, a commonly used model specifies that, conditional on a normal random effect, the marginals of the conditional distribution are given by the logistic link. In that situation, the likelihood function does not have a closed form and extensive numerical methods are needed.

Note that Equation (1) imposes the same correlation structure regardless of family relations. More accurate descriptions are possible by assuming different random effects for different family relations, but this increases the number of parameters to estimate. Petersen [7] discussed some random effect models for correlated life times. Similar structures can also be adapted for the dichotomous phenotypes. For ease of presentation and due to space limitation, we work with the simplified but illustrative Equation (1) here.

There have been some results on analysis of Equation (1). Conaway [8] proposed the log-log link and log-gamma random effect for correlated binary data. He focused on the case in which there are no covariates, but this model can be easily extended to accommodate covariates. Pulkstenis et al. [9] used the log-log link and log-gamma random effect in a case study of longitudinal binary data for pain relievers. Both of these papers focused on the maximum likelihood estimators (MLE) based on the marginal likelihood function.

For families of larger size, the likelihood function becomes increasingly more complicated. Contribution to the likelihood function from the *i*^th ^cluster has  terms. Also MLE may be sensitive to model misspecifications. Here we propose a new least-squares approach for testing *H*_0_:*β*_*m *_= 0. Note that the parameters *β *and *η *can be identified from the marginal mean response function, thus a natural and simple approach is to use the GEE based on Equation (2). We further observe that, for the cross products *y*_*ij*_*y*_*il*_, *j *≠ l, in the *i*^th ^family, we have

*E*(*y*_*ij*_*y*_il_) = *P*(*y*_*ij *_= 1, *y*_*il *_= 1) = *B*_*ijl *_(*η*, *β*),

where

*B*_*ijl*_(*η*, *β*) = {1 + exp(*η *+ *x*_*ij*_*β*) + exp(*η *+ *x*_*il*_*β*)}^-exp(-*η*)^.

Considering that *η *is involved in the random effect induced correlation among family members, it may be more efficient to work with *Y*_*i *_as well as cross products *y*_*ij*_*y*_*il*_. For the *i*^th ^family let *Z*_*i *_be the *k*_*i*_(*k*_*i *_+ 1)/2 × 1 vector consisting of *Y*_*i *_and the *k*_*i*_(*k*_*i *_- 1)/2 cross products *y*_*ij*_*y*_*il*_, *j *≠ *l*, *j*, *l *= 1, ..., *k*_*i*_. Let *m*_*i *_= *E*(*Z*_*i*_), and *V*_*i *_be the diagonal matrix with variance of the components of *Z*_*i *_on the diagonal. Then we define  as the minimizer of



For obtaining *V*_*i*_, *m*_*i*_, we have

*E*(*y*_*ij*_) = *P*(*y*_*ij *_= 1) = *A*_*ij*_(*η*, *β*),   (4)





with *A*_*ij*_(*η*, *β*), *B*_*ijl*_(*η*, *β*) defined previously. Note that  is asymptotically equivalent to the root of the estimating equation



where *∂m*_*i *_is the vector of partial derivatives of *m*_*i *_with respect to (*β*, *η*). In the above estimating equation the working covariance matrix is diagonal, and thus the estimating equation is always well defined. However, numerically it is more stable to use the least squares approach.

Once the estimators  are obtained, due to the asymptotic equivalence to the GEE approach, the covariance matrix of  can be estimated by the robust estimator

*V*_*β *_= *A*^-1^*BA*^-1 ^   (8)

with



where (*β*, *η*) are replaced by . A more stable but numerically more intensive alternative for estimating the covariance matrix is to use a bootstrapping method to resample family units a large number of times. Decompose  where  is the estimator for *β*_*m*_. Now the hypothesis *H*_0_:*β*_*m *_= 0 can be tested using the asymptotic normality of the *z *score based on .

Note that in the least-squares approach above, *β *can be interpreted as a regression parameter in the mean response function *EZ*_*i*_, which includes the cross product terms. We can similarly define a least squares estimator of (*β*, *η*) by working with *Y*_*i *_and its mean response function *EY*_*i*_, without the addition of the cross product terms. In that case, *β *would be interpreted as a regression parameter in the mean response function *EY*_*i*_. In various numerical studies, the addition of the cross product terms improves the efficiency for small and moderate samples sizes.

## Results

We applied the proposed approach to the data from COGA. The data provide alcoholism diagnosis on 1,614 individuals from 143 families. We focus on two distinct categories for the alcoholism diagnosis, "affected" as case (609 individuals) and "purely unaffected" as control (261 individuals). The preliminary genome scan carried out for linkage analysis using the microsatellite data identified a gene *ADH3 *on chromosome 4 as a candidate gene. We found 4 single-nucleotide polymorphisms (SNPs) (rs1036475, rs1491233, rs749407, rs980972), which are located in the physical map location of *ADH3 *genes from the Illumina SNPs data. Without correcting the correlated structure between family members, a logistic regression on these 4 SNPs suggested that rs1037475 and rs980972 were significant predictors (*p*-values of 0.0032 and 0.0284, respectively). Also, a quick look at the 2 × 2 table stratified by sex showed some differences. This led us to the consideration of using sex as a covariate. Assuming a recessive genetic model, the new least-squares method showed a significant association between rs1037475 and alcoholism, with a *p*-value of 0.013. Further, the analysis showed a significant sex effect with *p*-value < 0.001. Without using the cross product terms, the corresponding least squares method also showed a significant association between rs1037475 and alcoholism with a *p*-value of 0.002, and a significant sex effect with *p*-value < 0.001. The smaller *p*-value for the association between rs1037475 and alcoholism might be due to the fact that a common correlation was assumed among all family members for the 870 individuals and 143 families. Violation of this assumption does not affect the mean response function *Ey*_*ij *_but would introduce some bias in the mean response function *Ey*_*ij*_*y*_*il *_for the cross product terms. This in turn might reduce the power of the corresponding association test. When we restricted our analysis to the 499 siblings in 141 families, we still found a significant sex effect, with or without using the cross product terms. However, with the cross product terms, a significant association between rs1037475 and alcoholism was found; and without the cross product terms, no such association was established. In all cases with the reduced dataset, the *p*-value was smaller with the cross product terms than without them.

## Conclusion

In this article we have proposed a new test of association between dichotomous disease outcomes and genetic markers for family data. When applied to the data from COGA, this new approach indicated an association between SNP marker rs1037475 and alcoholism. This new approach has the flexibility of adjusting for covariates, and sex was a significant covariate in this analysis. The use of complementary log-log link function and the conjugate log-gamma random effect, rather than the more common combination of logistic link function and normal random effect, allowed us to obtain closed forms for the means and variances for the responses and their cross products. Using these quantities enables us to derive parametric estimators via the least squares approach that avoids the difficulty in the GEE approach created by singularity of the working correlation matrix. The least squares approach is more robust and computationally much simpler to implement than the likelihood approach.

Simulation studies also yielded evidence that the efficiency of the new approach is high and often its behavior on small samples is better than the more complicated likelihood-based approach.

## Abbreviations

COGA: Collaborative Study on the Genetics of Alcoholism

GEE: Generalized estimating equation

MLE: Maximum likelihood estimators

SNP: Single-nucleotide polymorphism

## Authors' contributions

SY was involved in the design of the study and statistical analysis, and drafted the manuscript. JJ and J-PL performed the statistical analysis and participated in revising the manuscript. ZF was involved in the design of the study and participated in revising the manuscript.

## References

[B1] Risch N (2000). Searching for genetic determinants in the new millennium. Nature.

[B2] Risch N, Merikangas K (1996). The future of genetic studies of complex human diseases. Science.

[B3] Whittaker JC, Morris A (2001). Family-based tests of association and/or linkage. Ann Hum Genet.

[B4] Witte JS, Gauderman W, Thomas D (1999). Asymptotic bias and efficiency in case-control studies of candidate genes and gene-environment interactions: basic family designs. Am J Epidemiol.

[B5] Liang KY, Zeger S (1986). Longitudinal data analysis using generalized linear models. Biometrika.

[B6] Slager SL, Schaid DJ, Wang L, Thibodeau SN (2003). Candidate-gene association studies with pedigree data: controlling for environmental covariates. Genet Epidemiol.

[B7] Petersen JH (1998). An additive frailty model for correlated life times. Biometrics.

[B8] Conaway MR (1990). A random effects model for binary data. Biometrics.

[B9] Pulkstenis EP, Ten Have TR, Landis JR (1998). Model for the analysis of binary longitudinal pain data subject to informative dropout through remediation. J Am Stat Assoc.

